# Radiofrequency and targeted ultrasound enhance natural hyaluronic production: a pilot porcine study

**DOI:** 10.1007/s40477-025-01083-y

**Published:** 2025-09-22

**Authors:** Jan Bernardy, Klaus Fritz, Carmen Salavastru, Rea Jarosova, Natalie Kralova

**Affiliations:** 1 Veterinary Research Institute, Hudcova 296/70, 621 00 Brno, Czech Republic; 2 Dermatology and Laser Center, Landau in der Pfalz, Germany; 3 2nd Department of Dermatology, Spitalul Clinic Colentina, Carol Davila University of Medicine and Pharmacy, Bucharest, Romania; 4 Department of Experimental Biology, Faculty of Science, Masaryk University, Brno, Czech Republic

**Keywords:** Hyaluronic acid, Radiofrequency, Targeted ultrasound, Facial rejuvenation, Fibroblast stimulation

## Abstract

**Background:**

Loss of skin elasticity and youthful appearance is closely linked to reduced hyaluronic acid (HA) levels in the dermis. While topical HA products offer temporary effects, they do not address the root cause of HA depletion.

**Objective:**

The present study aims to describe how to induce HA synthesis through fibroblast stimulation.

**Materials and Methods:**

Twelve sows were divided into two groups. One group (*n* = 9) received four consecutive Radiofrequency (RF) and Targeted Ultrasound (TUS) treatments, the other group (*n* = 3) received four RF treatments only. Samples were collected via punch biopsy from the treated area of each animal at the baseline, 1-month, and 2-month follow-up. Samples were then processed and prepared for enzyme-linked immunosorbent assay as well as hyaluronic acid binding protein with diaminobenzidine (HABP-DAP) staining, which allows for visualization of HA within tissue.

**Findings:**

The group receiving RF + TUS treatment has demonstrated an increase from an average of 83.0 µg/g at the baseline to 163.0 µg/g at the 2-month follow-up of HA concentration in the skin. Histology samples clearly demonstrate higher tissue density with an increase of brown pigment due to the HABP-DAP stain, representing HA in the skin. In comparison, the RF standalone treatment group has shown no significant increase in HA concentrations.

**Conclusion:**

Study results show no significant increase in HA production following the RF stand alone treatment. Whereas the RF + TUS treatment group induced a significant HA production response as well as histophysiological changes in the dermis.

## Introduction

The distinction between the “biological” age and the chronological age has proven useful in the treatment of age related disease [1]. Biological aging is a complex process that correlates with the loss of function and vitality of organs. These processes manifest with observable and measurable biomarkers, thus indicating the biological age of an individual [1, 2]. The skin is a barrier between the external and the internal, and so is at a constant exposure to environmental stress. The mechanism of skin aging consists of two independent, yet molecularly analogous processes. Intrinsically, it occurs as a molecular component of the normal aging process all organs undergo. The extrinsic component of skin aging (photodamage) is due to exogenous stressors that accelerate aging [3]. Photo-damage of the skin increases its biological age and perceived age [4]. In the context of a single individual, biological aging of the skin can exceed their chronological age, making the biological age a more accurate reflection on the skin health status. Evolutionary psychologists revealed that the condition of one’s skin advertises the individual’s perceived health [5]. Thus, it is no surprise that there is a large interest in reversing the aging of the skin and maintaining a youthful appearance.

The physiological role of hyaluronic acid (HA) has been well established [6]. Research indicates that the first signs of skin aging are a result of the insufficient amount of HA produced and secreted into the extracellular matrix (ECM) by fibroblasts [7]. Due to its unique water retention capability, HA serves as a primary source of hydration in the ECM of the dermis. There, along with elastin and collagen provides structural support of the skin. Youthful skin owes its integrity, turgor, and elasticity to high water content, which in turn is maintained by healthy ECM components [3]. Despite its relatively simple structure in comparison to other physiological molecules in its weight category (20–2000 kDa), it has been revealed that HA fulfills a wide repertoire of physiological roles [8]. As a key component of the ECM, production and secretion of HA is decreased in senescent and aged fibroblasts, compromising the skins’ structural integrity as well as the ability to combat reactive oxygen species [9]. Therefore, reinvigorating epithelial fibroblasts is postulated to be the key in fighting skin aging.

Topical delivery of HA increases its concentration in the epithelium and as such has become a popular component of commercially available cosmetic formulations [10]. Although it has been proven to be an effective method of rejuvenating the superficial layer of the skin, it fails to address the cause of diminishing HA concentrations within the skin [11]. In further efforts in rejuvenation, it has been demonstrated that application of mechanical or heat stress increases metabolic activity of fibroblasts, causing them to synthesize collagen and elastin [12–14] however, without any impact on the hyaluronic acid production. It has been hypothesized that applying both the mechanical and heat stress simultaneously could amplify the fibroblast response, inducing hyaluronic acid synthesis along with collagen and elastin.

As such, a novel technology has been developed to provide simultaneous mechanical and heat stress to epithelial fibroblasts non-invasively. The thermal component of the technology is the monopolar radiofrequency (RF) causing the dermis to uniformly heat between 40 to 42 °C. Its counterpart, the mechanical stimulation of fibroblast, is provided by targeted ultrasound (TUS). Focusing on the reticular dermis, a layer with highest fibroblast concentration. Initial investigational studies have shown that such combined technology may have a positive effect on the hyaluronic acid levels. Current study aims to verify or dispute these findings with the main objective to compare the simultaneous application of RF and TUS with the application of RF only and its effects on fibroblast activity related to synthesis of hyaluronic acid.

## Materials and methods

### Animal model

The Institutional Animal Care and Use Committee and the Ethics Committee for Animal Protection of the Ministry of Agriculture approved this investigational, single-center, two-arm animal study. It was supervised and performed by the Veterinary Research Institute (VRI, Brno, CZ), with a certification for Good Laboratory Practice. For the duration of the study, the animals were stabled in VRI. Certified veterinarian and veterinary staff dealt with animal handling and ensured animal well-being.

Twelve sows (*Sus scrofa f. domestica*, 60–80 kg) were divided into two groups. Both groups underwent four 20-min treatments delivered to the abdomen, spaced 2–3 days apart. One group (*n* = 9) was treated with simultaneous radiofrequency (RF) and targeted ultrasound (TUS) (Exion, BTL Industries Inc., Boston MA). The second group (*n* = 3) was treated by standalone monopolar RF. All utilized energies levels were set to 100%. The untreated side of the abdomen served as a control.

Propofol 2% MCT/LCT (Fresenius) at dosages of 1–2 mg/kg, delivered via cannula placed into the ear of the pigs, was used to induce general anesthesia for treatment procedure and sample collection. Preventive intubation and electrocardiogram (EKG) monitoring took place during general anesthesia. A certified veterinarian was overseeing the vital signs and assessing study-related adverse events and side effects. After the study completion, the animals were euthanized by an analgesic overdose (T61 a.u.v. inj, Intervet International B.V./MSD AH) administered by a veterinarian.

### Sample collection

Samples were taken with a 6–8 mm punch biopsy needle at the baseline (B), after the last therapy (T4), 1 month after the last treatment (M1) and 2 months after the last treatment (M2). The sample site was numbed by injecting local anesthetic (Lidocaine 2% a.u.v. inj, Fatro s.p.a., dosing 2 ml per biopsy) to relieve the pain upon waking up. The sampling wound was disinfected, enclosed with two clamps, and covered with a band-aid.

### Histology

The tissue samples obtained from the punch biopsy were fixed in a solution containing 10% neutral buffered formalin with 70% ethanol and 5% glacial acetic acid at room temperature for 48 h. Subsequently, the samples were rinsed in running tap water for 24 h, then gradually dehydrated using increasing concentrations of alcohol (70%, 80%, and 95%) for 3 h each, followed by three rounds of 100% alcohol for 45 min each. Next, the samples underwent clearing with three changes of xylen for 1 h each, followed by three paraffin immersions for 3 h per immersion. They were then embedded in paraffin blocks using HistoCore Arcadia H (Leica Biosystems, Germany). The prepared tissue blocks were sectioned to a thickness of 5 μm using a rotary microtome RM 2235 (Leica Biosystems) and placed onto SuperFrost Plus slides (Germany). The sections were allowed to dry at room temperature and subsequently stained using the HAPB-DAB method.

**Fig. 1:**
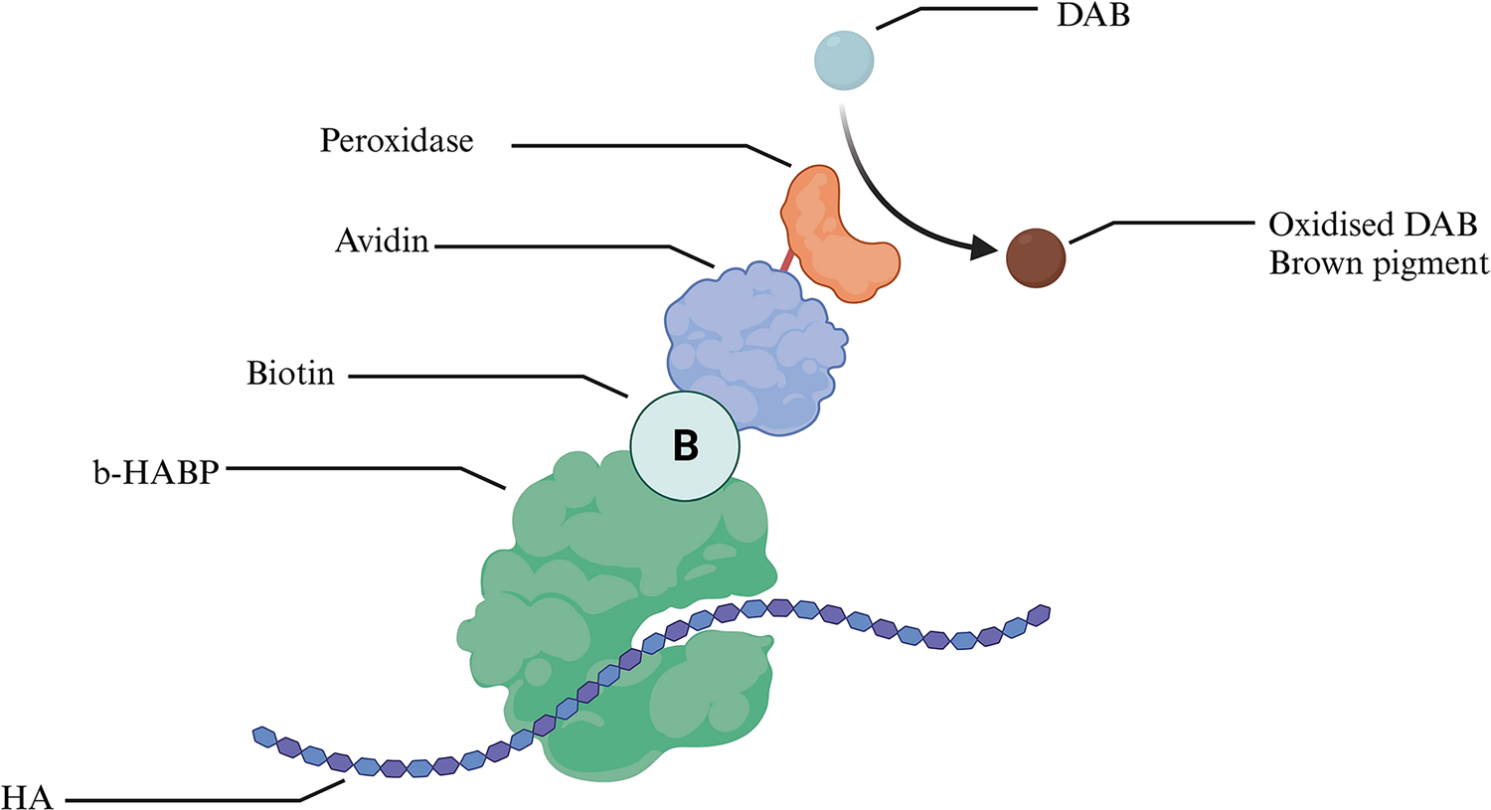
Diagram displaying the mechanism of action of HABP-DAB staining. Biotinylated Hyaluronic Acid Binding Protein (b-HABP) binds to HA. Biotin serves as a bridge connecting the Avidin-Peroxidase conjugate to form a b-HABP-Avidin-Peroxidase complex. Peroxidase oxidizes DAB into a brown pigment that is easily visualized with light microscopy. Diagram created with BioRender.com

Before staining, the slides were deparaffinized, rehydrated, and consequently blocked for 15 min with Avidin/Biotin Blocking Kit (SP-2001; Vector Labs, USA) in combination with Avidin D Solution followed by a PBS rinse, followed by 15 min Biotin Solution incubation and a PBS rinse. Slides were then incubated for 20 min with diluted normal goat blocking serum. HABP (Merck Millipore, USA) was diluted with normal goat serum (1:100) and incubated with the slides for an hour at room temperature. After the incubation, slides were washed in PBS 3 times. Slides were incubated for 30 min with Vectastain ABC Reagent (PK-6101 Rabbit IgG; Vector Labs), followed by a second wash. DAB substrate was added, and the slides were washed in distilled water and counterstained with Mayer hematoxylin (Penta, Czech Republic), clarified, dehydrated, and mounted. Stained samples were observed and photographed using an automated slide scanning microscope (Hitachi Axio Scan.Z1) in a bright field. HABP-DAB mechanism of action is described in Fig. 1.

### ELISA

In order to detect the HA in collected tissue, the samples were weighed (90–150 mg) and diced for better bead homogenization (MagNA Lyser, Roche Diagnostics, 7000 rpm, 2 × 60 s). After homogenization, Lysis Buffer (Thermo Fisher Scientific, USA) was added to the sample and was refrigerated at 4 °C overnight. The following day, the samples underwent another round of homogenization (7000 rpm, 2 × 60 s). Lysed samples were consequently centrifuged at 12,000 rpm for 20 min at 4 °C. The supernatant was removed and assayed immediately. In cases where assay could not be performed immediately, an aliquot was stored at − 80 °C until assayed. Commercial Hyaluronan Quantification Kit was used to evaluate the change in hyaluronic acid volume in subcutaneous tissue after the treatments in the animal model (amsbio, Cat. No. AMS.CSR-HA-96KIT).

### Statistics

ELISA results were analyzed using descriptive statistics (average, mean, standard deviation). Statistical significance was validated using Friedman’s test (*α* = 0.05) and Student *T*-test (*α* = 0.05).

## Results

### Histology

**Fig. 2:**
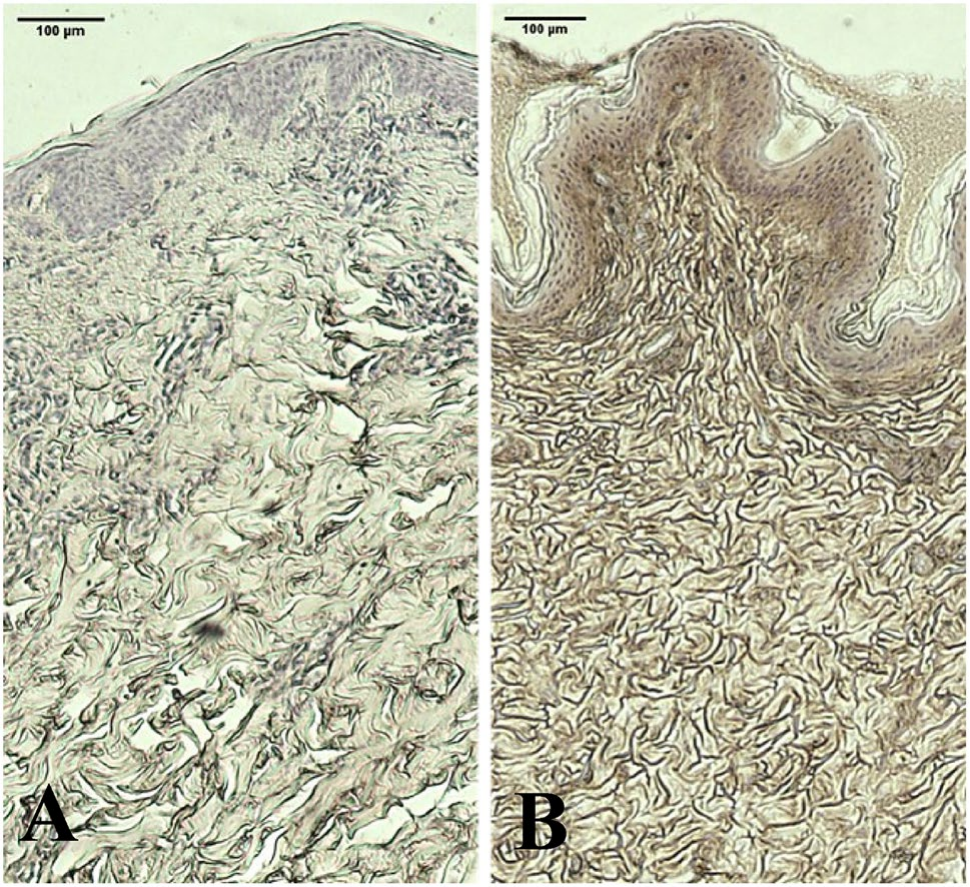
Histology images of the RF + TUS intervention group at the baseline (**A**) and the 2-month follow-up (**B**)

**Fig. 3:**
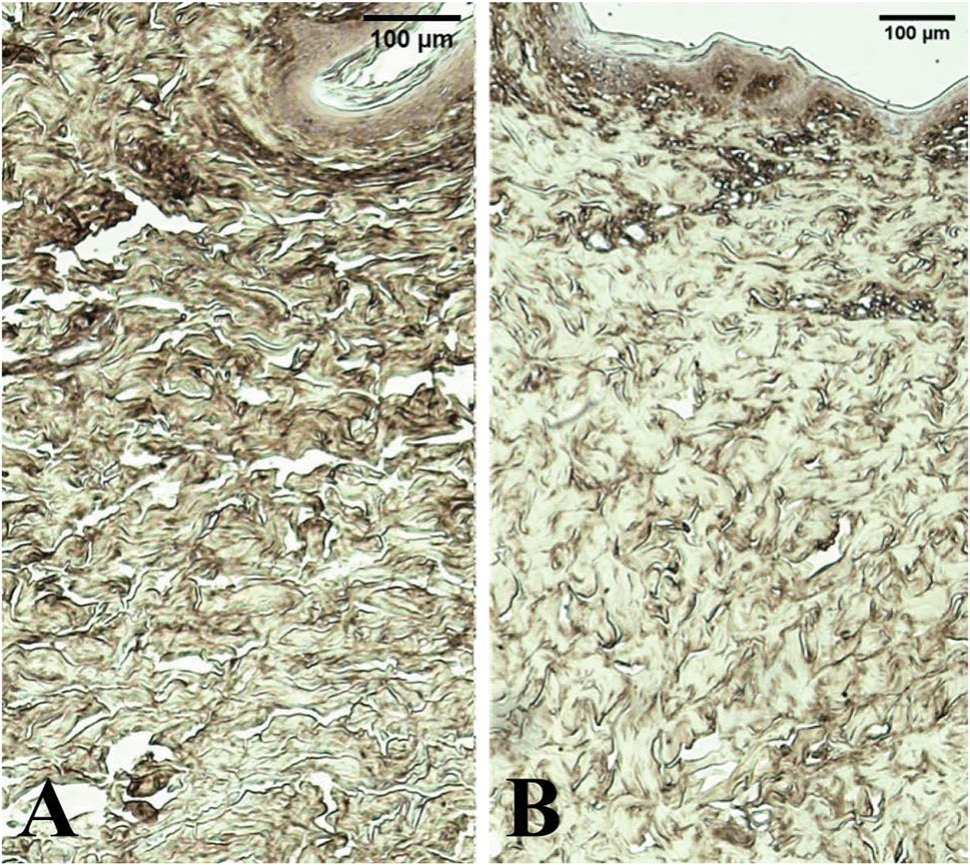
Histology images of the TF only group at the baseline (**A**) and the 2-month follow-up (**B**). Samples were processed with HAPB DAP staining to aid visualizing of HA (brown coloration)

Histological samples, stained with HABP-DAP show a visible increase of HA, as well as restructured ECM, clearly displaying higher overall density and increased integrity of the skin in the RF + TUS group as shown in Fig. 2. The RF only group displays no increase in pigmentation caused by HABP-DAP staining and no visible restructuring of the epidermis (Fig. 3).

Samples were processed with HAPB DAP staining to aid visualizing of HA (brown coloration). Note the higher intensity of the pigment as well as increased density of the skin on the right.

### ELISA

**Fig. 4:**
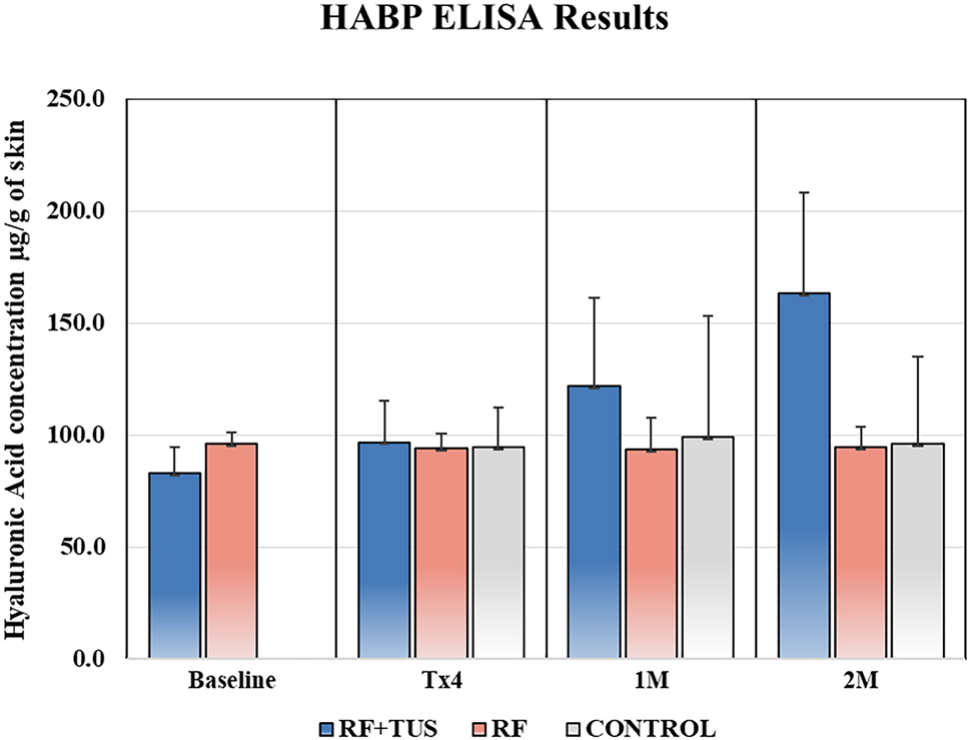
ELISA Results displaying hyaluronic acid levels found in tissue samples at the baseline, following the last therapy, at 1 and 2 month follow-up

ELISA HA assay shows that the intervention group (RF + TUS) had the highest benefit of the treatment (*p* < 0.0005), gradually raising the HA content to 163.0 ± 48.1 µg/g from 83.0 ± 12.3 µg/g of skin over the course of 2 months as per Fig. 4. The values in the RF only group and control samples oscillated at the baseline values without any significant change (*p* > 0.05) throughout the 2 months (Fig. 4). Student *T*-test shows no significant difference (*p* = 0.245) between the RF and RF + TUS groups at the baseline.

## Discussion

HA is synthesized and secreted by fibroblasts into the ECM. Despite a very high daily turnover rate; HA diminishes as we age [15]. Severely declining at the age of 47, it is estimated that by the age of 60 one can see a 50% decrease in HA concentration, reaching up to 75% reduction by the age of 70 [16]. This decrease in enzymic production of HA manifests as loss of skin elasticity, wrinkles and lines [3]. To accommodate for epidermal loss of HA, dermatological procedures have focused on replenishing the skin with HA via topical delivery, fillers and injections [10, 17]. However, these are temporary solutions as they do not address the cause of diminishing HA concentrations throughout the skin. It is important to clarify that, although both modalities attempt to restore the youthful looks, RF + TUS therapy in comparison to HA fillers has different mechanisms of action with different objectives. HA fillers and injections aim to restore volume lost due to aging or simply increase it for aesthetic purposes [18]. In the present study, we introduce a novel technology as a solution to depleted HA by endogenously rejuvenating the skin via thermal and mechanical stimulation and consequent activation of repair pathways, instead of applying a filler-like volumizing substance.

Physiological studies on fibroblasts conclude that external stress is a crucial component in increasing their activity [19]. Akin to muscle tissue, moderate mechanical stress (exercise, body movement) on the ECM strengthens it and reinforces its function [20]. Considering the external stress induced fibroblast stimulation, a number of studies have attempted to exogenously increase fibroblast metabolism. Heat and mechanical stress were the obvious choice. When applied independently, these external stimuli have shown to increase elastin synthesis by fibroblasts. However, there was no significant difference in HA production [21–23]. In their findings, the studies have shown that fibroblasts respond differently to heat and mechanical stress, respectively. Consequently, efforts have been made to apply both heat and mechanical stimuli simultaneously. The results of those studies have demonstrated synergistic effects of stressors on the fibroblast, leading to significant increase in hyaluronic acid production [24]. Following the course of this study, a clear trendline can be observed for the RF + TUS group; the HA continuously rises, peaking at the last sample collection. As indicated above, the heating stimulation of standalone RF was not able to induce a change in HA concentrations. Indicating the necessity for combining the technologies for the desired effect. Furthermore, previous studies have observed significant increase in neocollagenesis and neoelastinogenesis in addition to HA synthesis when applying the same technology [25, 26].

The limitations of this pilot study involve using the animal model. However, porcine skin has been established as analogous to human in many clinical studies [27–31]. The two methods used to evaluate the efficacy of the RF + TUS technology allowed us to quantise but also visualize the changes in HA biosynthesis. In consolidation, the previous study used additional methods in validating the increase of HA synthesis when combining RF + TUS technologies, such as: MALDI-TOF, confocal microscopy and PCR [24]. Future prospects for the study may involve a larger sample size and progression to the human model. Post-therapy sample collection at the 3rd and 6th month would allow for extension of the trendline and a better understanding of the dynamics of HA concentrations. Additionally, insight into quantitative and qualitative evaluation of the effects of RF + TUS on the other ECM components would be of great interest.

## Conclusion

In accordance with the past studies, we observe a statistically significant increase in the biosynthesis of HA within the ECM of the epidermis after RF + TUS therapy. There was no observable increase in HA synthesis with treatment utilizing RF only technology. In the context of here presented findings, we conclude that TUS in combination with RF is essential for inducing the physiological response causing the increase of HA in the skin. Validated by two different experimental approaches, we present a novel technology that is capable of not only restructuring the epidermis but effectively addresses HA depletion at its root cause. If confirmed effective on a larger scale, the RF + TUS technology would yield a dramatic shift in the aesthetic industry as a first non-invasive approach to boost the hyaluronic acid levels in the skin having far-reaching implications for skin rejuvenation.

## Data Availability

N/A.
